# Inhibitors of apoptosis proteins in human cervical cancer

**DOI:** 10.1186/1471-2407-6-45

**Published:** 2006-02-27

**Authors:** Magali Espinosa, David Cantú, Norma Herrera, Carlos M Lopez, Jaime G De la Garza, Vilma Maldonado, Jorge Melendez-Zajgla

**Affiliations:** 1 Subdirección de Investigación Básica. Instituto Nacional de Cancerología. Mexico City, Mexico; 2 Departamento de Ginecologia. Instituto Nacional de Cancerología. Mexico City, Mexico; 3 Escuela de Medicina, Instituto Politécnico Nacional, Mexico City, Mexico; 4 Research Division. Hospital Juarez de Mexico. Mexico City, Mexico

## Abstract

**Background:**

It has been shown that IAPs, in particular XIAP, survivin and c-IAP1, are overexpressed in several malignancies. In the present study we investigate the expression of c-IAP1, c-IAP2, XIAP and survivin and its isoforms in cervical cancer.

**Methods:**

We used semiquantitative RT-PCR assays to analyze 41 cancer and 6 normal tissues. The study included 8 stage I cases; 16 stage II; 17 stageIII; and a control group of 6 samples of normal cervical squamous epithelial tissue.

**Results:**

c-IAP2 and XIAP mRNA levels were similar among the samples, cervical tumors had lower c-IAP1 mRNA levels. Unexpectedly, a clear positive association was found between low levels of XIAP and disease relapse. A log-rank test showed a significant inverse association (*p *= 0.02) between XIAP expression and tumor aggressiveness, as indicated by disease relapse rates. There were no statistically significant differences in the presence or expression levels of c-IAP1 and c-IAP2 among any of the clinical variables studied. Survivin and its isoforms were undetectable in normal cervical tissues, in contrast with the clear upregulation observed in cancer samples. We found no association between survivin expression and age, clinical stage, histology or menopausal state. Nevertheless, we found that adenocarcinoma tumors expressed higher levels of survivin 2B and DeltaEx3 (*p *= 0.001 and *p *= 0.04 respectively, by Kruskal-Wallis). A multivariate Cox's partial likelihood-based analysis showed that only FIGO stage was an independent predictor of outcome.

**Conclusion:**

There are no differences in the expression of c-IAP2 and XIAP between normal vs. cancer samples, but XIAP expression correlate in cervical cancer with relapse of this disease in the patients. Otherwise, c-IAP1 was downregulated in the cervical cancer samples. The expression of survivin was upregulated in the patients with cervical cancer. We have found that adenocarcinoma presented higher levels of survivin isoforms 2B and DeltaEx3.

## Background

Life and death of cells must be balanced if tissue homeostasis is to be maintained. The main (though not the only) death mechanism by which mammalian cells maintain homeostasis is apoptosis. Dysregulation of apoptosis clearly contributes to the pathogenesis of various human diseases including cancer. Defects in the apoptotic pathway can eventually lead to expansion of a population of neoplastic cells and affect the intrinsic ability to respond to therapy.

Caspases, a group of cysteine proteases, are considered the central executioners of apoptosis. The important role of these proteins in cell death has generated intense research in order to find both positive and negative regulators of their activity. The physiological inhibitors of caspases are a group of antiapoptotic proteins termed IAPs, which are conserved across evolution, with homologues in both vertebrate and invertebrate animal species.

So far, eight human IAPs have been identified. Among these, XIAP (X-linked IAP) is the best-characterized. This protein is a potent suppressor of apoptosis owing to its ability to bind and inactivate caspases [1]. In addition to their caspase-inactivating properties, c-IAP1 and c-IAP2 are parts of a signaling complex that is recruited to the cytoplasmic domain of the type-2 Tumor Necrosis Factor Receptor (TNFR2) [2]. XIAP, c-IAP1 and c-IAP2 are thought to inhibit caspases 3, 7 and 9 directly [3]. Survivin, another member of the IAP family, is expressed during embryonic development but is absent from terminally differentiated adult tissues. This protein is prominently expressed in transformed cell lines and in many human tumors [4]. Survivin is structurally unique because unlike other IAPs it contains only a single BIR repeat and lacks the carboxyl-terminal RING domain. Its expression is regulated in a cell cycle-dependent manner with maximum levels occurring during the G2/M phase [5]. In cell culture systems, overexpression of survivin had been consistently associated with inhibition of cell death initiated by either the extrinsic or intrinsic apoptotic pathways [6], including those mediated by p53 [7] and exposure to antineoplastic agents [8]. In 1999, Mahotka *et al*. [9] described two novel alternatively-processed survivin transcripts, designated survivin-DEx3 (lacking exon 3) and survivin-2B (retaining part of intron 2 as a cryptic exon). Survivin-Dex3 retains its antiapoptotic function and survivin 2B shows a reduction of antiapoptotic potential compared to the type form.

Resistant tumors pose a serious problem in the treatment of cancer patients by antineoplastic agents. Although there is some controversy, accumulating experimental evidence supports the view that initial damage by chemotherapeutic agents converges into a common apoptotic pathway. In this regard, upregulation of IAP family members would certainly be advantageous for the tumors. Indeed, as data regarding different tumors accumulate, a widespread expression of IAPs, especially survivin, has been revealed [10]. Additionally, roles have been proposed for these proteins in cancer diagnosis and prognosis and even as therapeutic targets [11]. Nevertheless, the exact role of each IAP and their interplay in a particular cancer type are as yet unclear.

In the present study, we analyzed the expression of XIAP, survivin and its isoforms, c-IAP1 and c-IAP2 by means of RT-PCR assays in cervical cancer samples.

## Methods

### Cell lines and tumor samples

Cervical cancer cell line (HeLa) was obtained from American Type Culture Collection and cultured as monolayer in Dulbecco Modified Eagle Medium (DMEM) containing 10% (V/V) fetal bovine serum (GIBCO, Bethesda, MD, USA) at 37°C in a humidified atmosphere of 5% (V/V) CO_2_.

Cervical cancer samples were obtained from the Instituto Nacional de Cancerología, México. Written consent was obtained from patients before the samples were collected. Tumors were classified according to the International Gynecology and Obstetric Federation system (FIGO). Ninety consecutive patients were recruited in the gynecologic cancer clinic of the Institute. Due to failure of patients to continue treatment and be subjected to clinical follow-up, 31 were lost. In addition, 18 samples were unsuitable for analysis, due to insufficient cancer tissue in the histological control (see below). Thus, the study included only 41 samples: 8 IB; 16 IIB; 17 IIIB; and a control group, 6 samples of normal cervical squamous epithelial tissues. Histopathological grading was done according to the FIGO (International Federation of Gynecology and Obstetrics) classification system. The median follow-up was 31.2 months, with a range of 19 to 57.7 months. Samples were assessed and only those which presented more than 90% neoplastic cells were included. Forty-three percent of the patients received radiotherapy, and the rest received radiotherapy and chemotherapy.

Normal cervical tissue samples were used as controls and were obtained after hysterectomy performed for benign conditions such as myomatosis. All tissue samples were used only after confirmation their malignancy or benignity by a gynecological pathologist at the Surgical Pathology Department of the Institute.

### RNA isolation and RT-PCR

Extraction and RT-PCR analysis were performed as described previously [12]. Briefly, total RNA was extracted from cell lines, tumoral and non-neoplastic tissue samples with Trizol reagent (Invitrogen) following the manufacturer's protocol. RNA purity was confirmed by the 260/280 nm ratio and its integrity was established by agarose gels. Total RNA (2 μg) was reverse-transcribed in a final 20 μl reaction volume using 15 U ThermoScript reverse transcriptase, 2.5× RT Buffer and random hexamers (ThermoScript RT-PCR, Invitrogen). PCR reactions contained 0.25 μl amplitaq gold polymerase (Applied Biosystems, ROCHE), 2.5 μl 10× reaction buffer, 0.5 μl dNTP mixture (10 mM), 1 μl sense primer (10 μM), 1 μl anti-sense primer (10 μM) and 1 μl cDNA in a 25 μl final volume. Survivin primers were: sense 5' GCCATGAATTCATGGGTGCCCCGACGTTGC 3' and anti-sense 5' AGCTCTCTAGAGAGGCCTCAATCCATGGCA 3'. These primers are also able to amplify the survivin isoforms 2B and DelatEx3. XIAP primers were: sense 5' GCACGAGCAGGGTTTCTTTATACTGGTG 3' and antisense 5' CTTCTTCACAATACATGGCAGGGTTCCTC 3'. c-IAP1 primers were: sense 5' GAATACTCCCTGTGATTAATGGTGCCGTGG 3' and antisense 5' TCTCTTGCTTGTAAAGACGTCTGTGTCTTC 3'. c-IAP2 primers were: sense 5' GAATACTCCCTGTGATTAATGCTGCCGTGG 3' and antisense 5' TCTCTTGCTTGTAAAGACGTCTGTGTCTTC 3'. GAPDH primers were: sense 5' CCCCTTCATTGACCTCAACT 3' and antisense 5' TTGTCATGGATGACCTTGGC 3'. RT-PCR steps were 25° 10', 50°C 50' and 85°C 5'. To corroborate the specificity of amplification, the PCR products were electrophored, excised from the gel and sequenced.

The products were electrophoresed in 1% agarose gels stained with ethidium bromide. A curve was generated for each sample to verify that the amplification reactions proceeded logarithmically. The PCR products were normalized to those obtained from GAPDH (mRNA) amplification, used as internal reference gene and referred to a HeLa cDNA standard for each gel. Gene expression measurements were repeated at least twice. To corroborate the specificity of amplification, the PCR products were excised from the gel and sequenced.

### Statistical analysis

To detect associations between pathological tumor parameters and normalized IAP expression we used Kruskal-Wallis test. Expression groups for XIAP and Survivin were defined using the 50^th ^percentile of the corrected semiquantitative RT-PCR data between each variable and the categorized variable. Survival was evaluated by Kaplan-Meier curves for patients with each variable and the curves were compared by a log rank test. Statistical significance was inferred when the *p *value was less than 0.05. To determine the influence of multiple variables simultaneously, a multivariate Cox proportional hazards model was applied to the clinical variables and IAPs expression data. Wald's test was used to determine statistical significance in the Cox models. The statistical package SPSS 12.0 was used for these analyses.

## Results and Discussion

**Figure 1:**
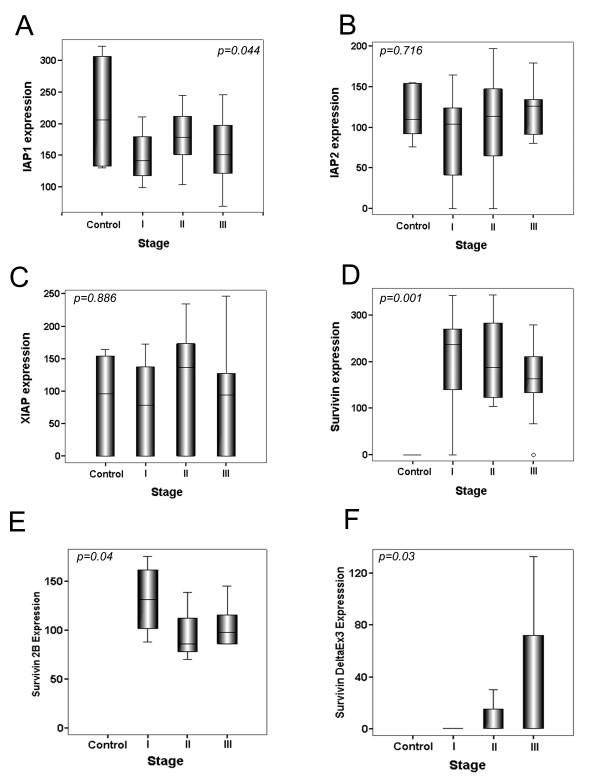
Relative expression of IAP mRNAs, determined by semiquantitative RT-PCR in cervical cancer samples at different stages and control normal cervical samples. *a*) c-IAP1 expression; *b*) c-IAP2 expression; *c*) XIAP expression; *d*) Survivin expression *e*) Survivin 2B expression *f*) Survivin DeltaEx3 expression. Procedures were performed as described in "Materials and Methods".

**Figure 2:**
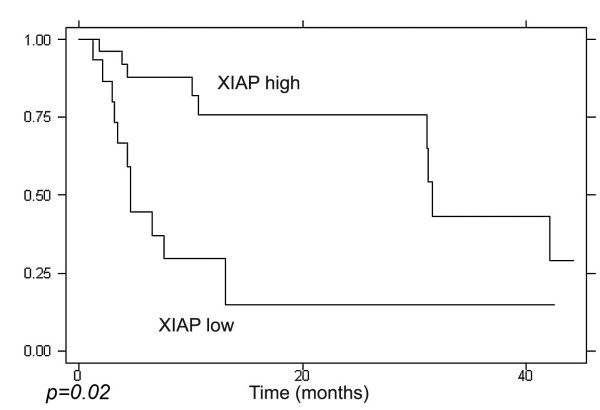
Kaplan-Meier survival estimates in cervical cancer patients by XIAP expression. Patients expressing low levels of XIAP versus high levels of XIAP, based on the 50^th ^percentile, were plotted for relapse over the time shown (months). Statistical analysis was performed by a log rank test.

In clear contrast to reports showing upregulation of several IAPs in tumors such as lung and prostate, the IAP-2 and XIAP mRNA levels were similar among control and cancer tissues (Fig. 1). There were no differences between clinical variables, IAPs levels and therapy received. Interestingly, cancer samples showed even lower c-IAP-1 mRNA levels than normal tissues (Median 205.51 ± 79.6 vs 161.22 ± 44.8, p= 0.04 by Kruskal-Wallis). There were no statistically significant differences in the presence or expression levels of c-IAP-1 and c-IAP-2 among any of the clinical variables studied, including age, clinical stage, histology, or menopausal state (Tables 1 and 2). Similarly, no differences were found between XIAP expression and stage, menopause or histology of the tumors (Table 3) Nevertheless, a clear association was found between low expression of XIAP and disease relapse (Fig. 2; log-rank *p *= 0.02).

**Figure 3:**
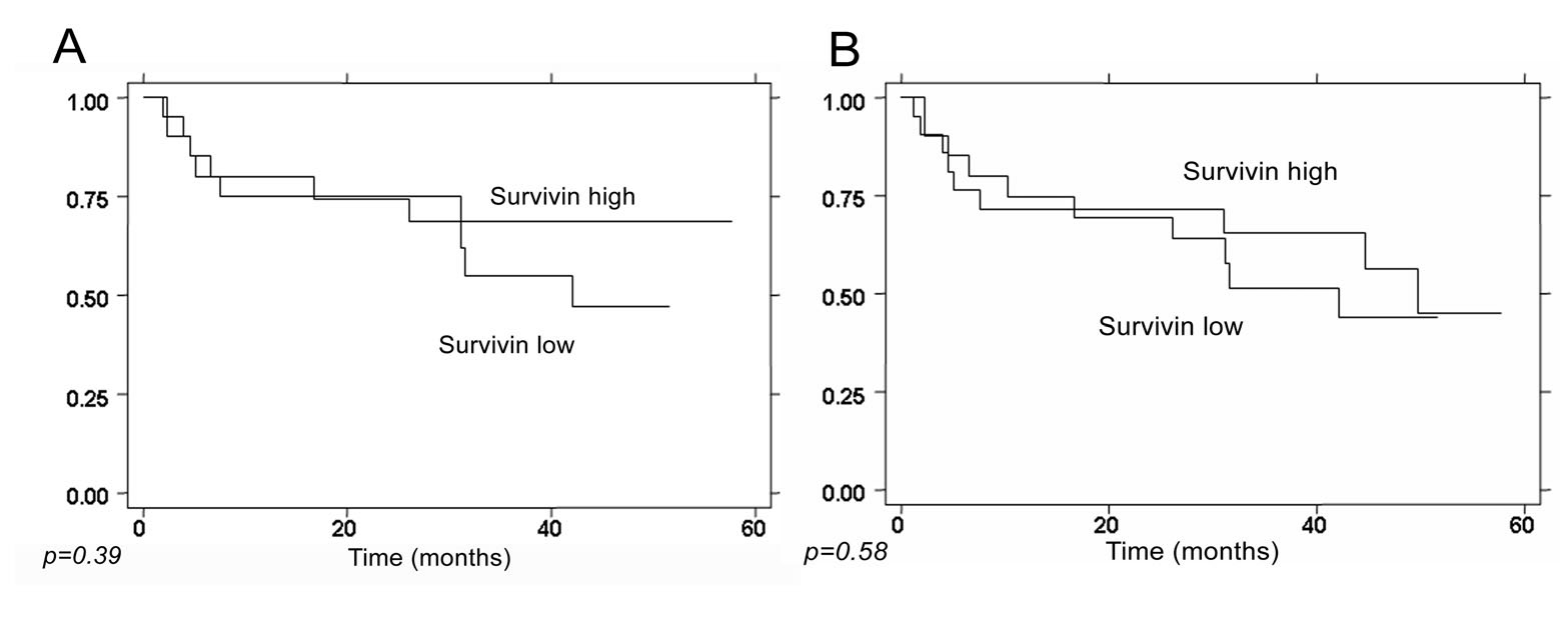
Kaplan-Meier survival estimates in cervical cancer patients by Survivin expression. Patients expressing low levels of Survivin versus high levels of Survivin, based on the 50^th ^percentile, were plotted for A) Death and B) Relapse over the time shown (months). Statistical analysis was performed by a log rank test.

While survivin is undetectable in normal, fully differentiated tissues, high expression levels are observed in a broad range of malignancies [4]. In the present report, survivin and its isoforms were undetectable in normal cervical tissues, in contrast to the clear upregulation observed in cancer samples (Fig. 1). High levels of survivin, which correlated with clinical status, have been reported for several malignancies [13]. We found no association between survivin expression and clinical stage, histology or menopausal state (Table 4). Similarly, no prognostic association was found between survivin expression and disease relapse or death (Fig. 3). Nevertheless, we found that even when similar numbers of patients in the different clinical stages expressed survivin isoforms, patients afflicted with the clinically more aggressive adenocarcinoma tumors expressed higher survivin 2B and DeltaEx3 levels (*p *= 0.001 and *p *= 0.04 respectively, by Kruskal-Wallis) (Table 5 and 6). When a multivariate Cox's partial likelihood-based analysis was applied to the data, only FIGO stage was an independent predictor of outcome (Table 7).

The inhibitors of apoptosis proteins (IAPs) are a family of antiapoptotic proteins that share the BIR (Baculoviral IAP Repeat) motif. These proteins bind and inhibit caspases 3, 7 and 9 and also modulate cell division, cell cycle progression and signal transduction pathways. In this regard, it has been shown that tumor cells have high intrinsic levels of apoptotic signaling and need proteins that inhibit apoptosis to counterbalance this [14]. In addition, several studies have demonstrated upregulation of certain IAPs in response to chemotherapy, mediating cell resistance to apoptosis [12]. For these reasons, several authors have explored the possible use of IAPs as early diagnostic or prognostic markers, and even as possible targets for malignancy treatment. For example, IAPs such as survivin are being investigated as diagnostic markers for the presence of occult malignancy [10]. In addition, IAP overexpression is a poor prognostic marker for a variety of solid tumors and hematological malignancies [15].

In the present study we found that in cervical cancer, most of the IAPs analyzed were not overexpressed, with the notable exception of survivin. These results are consistent with the previous immunohistochemistry report of Liu *et al*. [16], who found no differences in c-IAP-2 and XIAP levels between normal and cancerous cervical samples. Unexpectedly, we found a significant downregulation of c-IAP1 in tumor samples. This change of expression could be caused by a mechanism compensating for the increased survivin level. It has been shown that c-IAP-1 interferes with the function of survivin in mitosis [17], so it is not unreasonable to propose that concurrent expression of both proteins is not favored during carcinogenesis. In addition, a previous report that c-IAP1 and 2 are upregulated in XIAP-deficient mice [18] supports the idea of a compensatory mechanism. Alternatively, differences in signal transduction cascades in cervical cancer could account for the differences. One of the main IAP expression regulators is the pleiotropic transcription factor NF-kappa B. This factor has emerged as a major culprit in a variety of human cancers mainly because of its ability to protect transformed cells from apoptosis. This protection is, at least in part, afforded by the upregulation of IAPs [19]. Heterogeneity in the components and transduction signaling activation of this transcription factor has been reported for specific breast cancer subgroups [20], so it is plausible that in cervical cancer, differences in NF-kappa B complexes could account for the lack of c-IAP1 and 2 overexpression.

It is noteworthy that, in contrast to most recent reports on other cancer types, we found a positive role for XIAP as a prognostic factor. A log-rank test showed a significant inverse association (*p *= 0.02) between XIAP expression and tumor aggressiveness as indicated by the rate of relapse. This unexpected result could be due to differences in mRNA and protein levels in particular tissue types. Indeed, it has been shown that the translation of XIAP is a highly regulated process [21]. As stated previously, IAPs expression shows remarkable plasticity, so it is not unlikely that differences in mRNA and protein levels could have caused this result by means of a negative feedback on transcription. Further immunohistochemical analyses are underway in our laboratory to elucidate this matter.

Survivin is unique among IAPs in its ubiquitous expression during development and its absence from most normal adult tissues [4]. Its cell cycle-regulated expression at mitosis and association with the mitotic apparatus are related to the faithful segregation of sister chromatids and timely separation of daughter cells. Survivin is re-expressed in most cancers and associated with tumor aggression and decreased patient survival rates [22], making it an attractive diagnostic and therapeutic target. For example, associations between high levels of survivin and clinical status and/or prognosis have been reported in colorectal cancer [23], among others. It has been shown that survivin expression increases during cervical carcinogenesis, following a gradient from low-grade squamous intra-epithelial lesions to high-grade squamous epithelial lesions and squamous tumors [24]. In the present study, we found no association between survivin expression levels and any clinical variable, as Lee *et al*. [25] reported on the basis of immunohistochemical evidence. Similar results were found in breast cancer [26], where survivin expression did not correlate with tumor stage, histological stage or nodal status; and in osteosarcoma and transitional cell cancer [27]. Nevertheless, in the first two studies, a positive association was found between survivin immunolocalization and prolonged survival. It seems that not only the expression level but also the specific location of the protein is important for the participation of survivin in carcinogenesis. This pattern of sub-localization and its relevance to cervical cancer warrant further study.

Of clear interest was our finding that survivin isoforms were expressed predominantly in adenocarcinomas. Compared to squamous cell carcinoma, cervical adenocarcinoma has poorer prognosis, metastasizes earlier to lymph nodes and is relatively resistant to radiotherapy [28]. Higher levels of survivin 2B and deltaEx 3 were found in adenocarcinoma samples, although there were no prognostic implications for this upregulation. Our results concur with recent reports about breast cancer [29], where both isoforms were upregulated, and in brain tumors [13], where survivin deltaEx 3 was also upregulated. It is interesting to note that survivin 2B, which has reduced antiapoptotic function [9], correlates negatively with tumor stage, histological type and depth of tumor invasion in gastric cancer [30]; although this is not universal, as stated previously. Additionally, it has been found that an increased staining for survivin was found in cervical adenocarcinoma versus squamous cell carcinoma [31]. Since the antibodies used in this study do not discriminate between survivin isoforms, the results could be reflecting the increase of total survivin levels, including all the known isoforms, as reported here. These data suggest that careful consideration must be given to the relative expression levels of the survivin splice isoforms in tumors. We were not able to assess the prognostic significance of these results because of our small sample size, but further analyses are warranted by these preliminary findings.

## Conclusion

It has been shown that IAPs, in particular XIAP, survivin and c-IAP1, are overexpressed in several malignancies. In the present report, we analyze the expression levels of these proteins in cervical cancer by means of RT-PCR assays. Two main conclusions derive from our results: first, a clear positive association was found between low levels of XIAP and disease relapse. The second, expected from published results in other tumors, was the survivin and its isoforms upregulation observed in cancer samples. We found that adenocarcinoma tumors expressed higher levels of the survivin 2B and DeltaEx3 isoforms. Unfortunately, Cox multivariate analysis showed that only FIGO stage is an independent prognostic factor in cervical cancer.

IAPs are attractive therapeutic targets, and efforts are underway to develop antisense, vaccines and chemical IAP inhibitors that may be useful for treating a variety of malignancies [32-36]. Knowing the expression profiles of IAPs in specific tumor types will undoubtedly help in designing strategies to this end. Despite these potential clinical applications, however, the challenge remains to incorporate the findings into actual clinical practice.

## Competing interests

The author(s) declare that they have no competing interests.

## Authors' contributions

ME carried out the molecular analysis and helped with the statistical analysis, DC participated in the design of the study and in the analysis of the clinical variables, NH participated in the design of the study and RNA extraction. CL contributed with the interpretation of data and clinical assessment of the results. JDlG participated in the design of the study and carried out the statistical analysis. VM drafted the paper, participated in the design of the study and helped with the molecular analysis, JMZ conceived of the study, participated in its design and coordinated it. All authors read and approved the final manuscript.

## Pre-publication history

The pre-publication history for this paper can be accessed here:



## Figures and Tables

**Table 1:** c-IAP1 expression in cervical cancer samples.

**Variable**	No. patients (n = 41)*	Median c-IAP1 expression	*AD*	*p*
**FIGO stage**				
IB	8 (8)	141.23	40.14	0.14
IIB	16 (16)	178.02	39.80	
IIIB	17 (17)	151.29	48.73	
**Histology of tumors**				
Squamous cell	34 (34)	160.14	45.80	0.09
Adenocarcinoma	5 (5)	198.01	20.93	
Adenosquamous	2 (2)	135.45	22.40	
**Menopause**				
Pre-Menopause	10 (10)	148.69	34.33	0.61
Menopause	8 (8)	169.87	43.47	
Post-Menopause	23 (23)	171.78	49.97	

*Number of patients expressing c-IAP1 versus total patients in the respective group (in parenthesis). AD: absolute median deviation. Kruskall-Wallis test used to test for differences.

**Table 2:** c-IAP2 expression in cervical cancer samples.

**Variable**	No. patients (n = 41)	Median c-IAP2 expression	*AD*	*p*
**FIGO stage**				
IB	7 (8)	104.02	59.25	0.48
IIB	14 (16)	113.58	60.73	
IIIB	17 (17)	125.55	28.42	
**Histology of tumors**				
Squamous cell	34 (34)	113.58	54.00	0.40
Adenocarcinoma	5 (5)	121.78	12.44	
Adenosquamous	2 (2)	103.97	33.79	
**Menopause**				
Pre-Menopause	5 (10)	120.74	28.19	0.88
Menopause	5 (8)	102.65	43.70	
Post-Menopause	23 (23)	121.78	58.16	

*Number of patients expressing c-IAP2 versus total patients in the respective group (in parenthesis). AD: absolute median deviation. Kruskall-Wallis test used to test for differences.

**Table 3:** XIAP expression in cervical cancer samples.

**Variable**	No. patients (n = 41)*	Median XIAP expression	*AD*	*p*
**FIGO stage**				
IB	5 (8)	78.14	71.34	0.66
IIB	10 (16)	136.54	93.26	
IIIB	11 (17)	93.96	78.65	
**Histology of tumors**				
Squamous cell	23 (34)	112.87	83.94	0.14
Adenocarcinoma	3 (5)	59.60	55.79	
Adenosquamous	0 (2)	0	0	
**Menopause**				
Pre-Menopause	5 (10)	29.80	93.51	0.61
Menopause	5 (8)	138.25	77.86	
Post-Menopause	16 (23)	103.52	81.19	

*Number of patients expressing XIAP versus total patients in the respective group (in parenthesis). AD: absolute median deviation. Kruskall-Wallis test used to test for differences.

**Table 4:** Survivin expression in cervical cancer samples.

**Variable**	No. patients (n = 41)	Median Survivin expression	*AD*	*p*
**FIGO stage**				
IB	5 (8)	236.74	107.26	0.34
IIB	10 (16)	187.67	88.25	
IIIB	11 (17)	163.35	71.12	
**Histology of tumors**				
Squamous cell	23 (34)	186.10	91.47	0.18
Adenocarcinoma	3 (5)	162.37	54.84	
Adenosquamous	0 (2)	164.04	93.16	
**Menopause**				
Pre-Menopause	5 (10)	165.07	75.41	0.88
Menopause	5 (8)	142.94	92.27	
Post-Menopause	16 (23)	210.42	85.46	

*Number of patients expressing Survivin versus total patients in the respective group (in parenthesis). AD: absolute median deviation. Kruskall-Wallis test used to test for differences.

**Table 5:** Survivin 2B expression in cervical cancer samples.

**Variable**	No. patients (n = 41)*	Median Survivin 2B expression	*AD*	*p*
**FIGO stage**				
IB	1 (8)	0.01	31.09	0.95
IIB	3 (16)	0.02	30.02	
IIIB	3 (17)	0.05	38.15	
**Histology of tumors**				
Squamous cell	3 (34)	0.01	21.56	0.001
Adenocarcinoma	4 (5)	86	43.82	
Adenosquamous	0 (2)	0	0	
**Menopause**				
Pre-Menopause	2 (10)	0.03	32.58	0.70
Menopause	2 (8)	0.02	40.27	
Post-Menopause	3 (23)	0.1	31.94	

*Number of patients expressing Survivin 2B versus total patients in the respective group (in parenthesis). AD: absolute median deviation. Kruskall-Wallis test used to test for differences.

**Table 6:** Survivin DeltaEx3 expression in cervical cancer samples.

**Variable**	No. patients (n = 41)*	Median expression Survivin DeltaEx3	*AD*	*p*
**FIGO stage**				
IB	0 (8)	0	0	0.59
IIB	2 (16)	0.09	24.51	
IIIB	2 (17)	0.11	35.68	
**Histology of tumors**				
Squamous cell	2 (34)	0.01	16.99	0.04
Adenocarcinoma	2 (5)	0.22	60.12	
Adenosquamous	0 (2)	0	0	
**Menopause**				
Pre-Menopause	2 (10)	0.14	48.94	0.33
Menopause	1 (8)	0.02	10.65	
Post-Menopause	1 (23)	0.01	14.99	

*Number of patients expressing Survivin DeltaEx3 versus total patients in the respective group (in parenthesis). AD: absolute median deviation. Kruskall-Wallis test used to test for differences.

**Table 7:** Analysis of maximum likelihood estimates under Cox's model.

**Factor**	**Hazard ratio (95% CI)**	***p***
(A) Univariate analysis of disease-free survival		
FIGO stage	2.41 (1.10–5.00)	0.018
Age	1.00 (0.97–1.00)	0.075
Menopause	0.77 (0.46–1.31)	0.063
Histology	2.58 (1.24–5.41)	0.011
Survivin	0.99 (0.99–1.00)	0.295
Survivin2B	1.00 (0.99–1.01)	0.592
SurvivinΔEx3	1.00 (0.99–1.02)	0.187
XIAP	0.69 (0.59–0.94)	0.048
c-IAP1	0.99 (0.98–1.01)	0.334
c-IAP2	0.98 (0.96–1.00)	0.115
(B) Multivariate Cox model of disease-free survival		
FIGO stage	3.13 (1.11–8.81)	0.031
Age	1.06 (0.98–1.13)	0.108
Menopause	0.29 (0.07–1.20)	0.089
Histology	1.77 (0.71–4.41)	0.071
Survivin	0.97 (0.99–1.00)	0.374
Survivin2B	1.00 (0.07–1.03)	0.743
SurvivinΔEx3	0.99 (0.07–1.02)	0.882
XIAP	0.79 (0.69–0.96)	0.516
c-IAP1	0.99 (0.97–1.01)	0.548
c-IAP2	0.99 (0.98–1.00)	0.486
